# Spin-coupling-induced Improper Polarizations and Latent Magnetization in Multiferroic BiFeO_3_

**DOI:** 10.1038/s41598-017-18636-9

**Published:** 2018-01-10

**Authors:** Hyun Myung Jang, Hyeon Han, Jung-Hoon Lee

**Affiliations:** 1 Department of Materials Science and Engineering, and Division of Advanced Materials Science, Pohang University of Science and Technology (POSTECH), Pohang, 37673 Republic of Korea; 2 Department of Physics, University of California, Berkeley, Berkeley, CA 94720-7300 USA

## Abstract

Multiferroic BiFeO_3_ (BFO) that exhibits a gigantic off-centering polarization (OCP) is the most extensively studied material among all multiferroics. In addition to this gigantic OCP, the BFO having *R3c* structural symmetry is expected to exhibit a couple of parasitic improper polarizations owing to coexisting spin-polarization coupling mechanisms. However, these improper polarizations are not yet theoretically quantified. Herein, we show that there exist two distinct spin-coupling-induced improper polarizations in the *R3c* BFO on the basis of the Landau-Lifshitz-Ginzburg theory: Δ*P*_*LF*_ arising from the Lifshitz gradient coupling in a cycloidal spin-density wave, and Δ*P*_*ms*_ originating from the biquadratic magnetostrictive interaction. With the help of *ab initio* calculations, we have numerically evaluated magnitudes of these improper polarizations, in addition to the estimate of all three relevant coupling constants. We further predict that the magnetic susceptibility increases substantially upon the transition from the bulk *R3c* BFO to the homogeneous canted spin state in a constrained epitaxial film, which satisfactorily accounts for the experimental observation. The present study will help us understand the magnetoelectric coupling and shed light on design of BFO-based materials with improved multiferroic properties.

## Introduction

**Figure 1:**
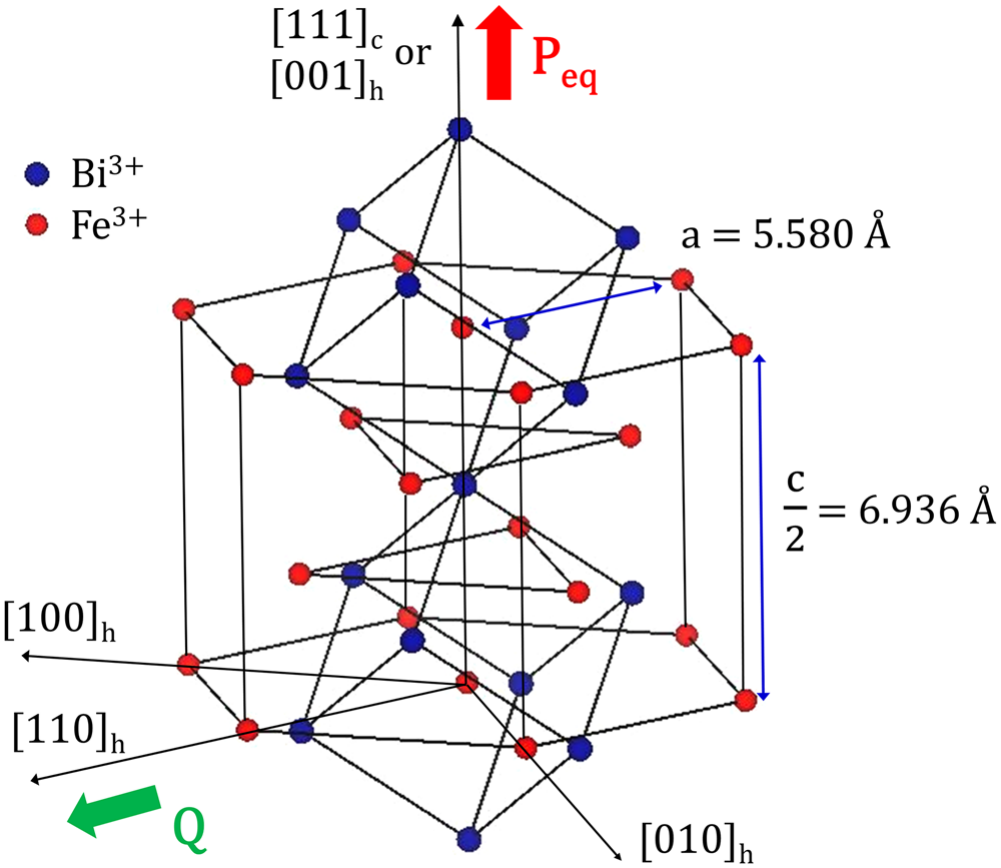
A half unit-cell hexagonal structure of BiFeO_3_ having *R3c* space-group symmetry. The hexagonal lattice parameters shown in the figure (*a* = *b* = 5.580 Å, *c* = 13.872 Å) are based on our previous Rietveld refinement^17^. In the same figure, a pseudo-cubic representation is also shown along the [111]_c_ direction which is equivalent to the polar [001]_h_ direction in hexagonal notation.

Multiferroics are an interesting group of materials that exhibit both ferroelectricity and anti-ferromagnetism with coupled electric and magnetic order parameters^1,2^. Multiferroism is the subject of intensive scientific investigations as these materials are able to offer a wide range of interesting applications that include sensors, transducers, memories, spintronics and ferroelectric photovoltaics^3–9^. Among numerous multiferroics, BiFeO_3_ (BFO) is currently the only ABO_3_-type simple perovskite that exhibits room-temperature multiferroism and, thus, is considered to be the most promising candidate for practical applications of multiferroics. It is a rhombohedrally distorted ferroelectric perovskite (*T*_*c*_ ≈ 1100 *K*) with the space group *R3c* and shows canted antiferromagnetism up to 643 K (Néel temperature, *T*_*N*_)^10–12^. The *R3c* BFO is known to possess the largest reported value of the switchable polarization of ~90 *μC*/*cm* ^2^ along the pseudo-cubic [111]_c_ direction (or equivalently, [001]_h_ in hexagonal notation; Fig. 1)^13,14^. According to the previous *ab initio* studies^14–17^, the stereochemically active lone-pair electrons originating from the hybridization of 6*s* and 6*p* atomic orbitals of Bi are responsible for the off-centering displacement (OCD) of the Bi ion along [111]_c_ (or [001]_h_). Interestingly, the *R3c* BFO is further characterized by the incommensurate cycloidal spin structure with a periodicity of 620 Å along the [110]_h_ direction in hexagonal setting^18,19^.

The magnetoelectric (ME) coupling between polarization (*P*) and magnetization (*M*) order parameters is the single most important subject of multiferroics^2^. In case of the *R3c* BFO, strong ME coupling is not anticipated due to the absence of a strong interaction between the ferroelectric OCD at the Bi-ion site and the *G*-type antiferromagnetic (AFM) spin moment at the Fe-ion site. However, the *G*-type AFM alignment plus the cycloidal spin ordering of BFO allows opportunities for interfacial magnetic coupling in multiferroic heterostructures, where BFO plays some important roles as ferroelectric substrate, AFM pinning layer for exchange bias, and interfacial quantum modulation donor^2^. Indeed, Saenrang and co-workers^20^ recently demonstrated deterministic and robust room-temperature exchange coupling between the BFO AFM order and the Co overlayer with ~90° in-plane Co-moment rotation upon single-step ferroelectric switching of the monodomain BFO. This has important consequences for practical, low power non-volatile ME devices utilizing BFO^20^.

In spite of extensive studies on the *R3c* BFO, however, possible ME coupling mechanisms and associated improper polarizations are not yet quantitatively resolved or lucidly explained. Herein, we show unequivocally that there exist two distinct spin-coupling-induced improper polarizations in the *R3c* BFO on the basis of the Landau-Lifshitz-Ginzburg phenomenological theory. These are: (i) a small parasitic improper polarization (Δ***P***_*LF*_) originating from the Lifshitz exchange coupling, which can be equated with the ***S***_*i*_ × ***S***_*j*_-type vector coupling induced polarization (Δ***P***_*DM*_) caused by the reverse Dzyaloshinskii-Moriya (DM) interaction, and (ii) a ***S***_*i*_ · ***S***_*j*_-type scalar coupling induced polarization (Δ***P***_*ms*_) caused by the biquadratic magnetostrictive exchange interaction. With the help of *ab initio* density-functional theory (DFT) calculations, we have evaluated magnitudes of these improper polarizations, in addition to the numerical estimate of all three relevant ME coupling constants. We have further predicted that the magnetic susceptibility increases substantially upon the transition from the bulk *R3c* BFO to the homogeneous canted spin state in a constrained epitaxial film. These studies will help us comprehensively understand the ME coupling mechanisms in the *R3c* BFO and shed light on design of BFO-based materials with improved multiferroic properties.

## Theoretical Analysis

### Improper Polarization and Invariant caused by the Reverse DM Interaction

As mentioned previously, the *R3c* BFO is represented by a pseudo-cubic unit cell with its proper polarization along the cubic [111]_c_ direction (i.e., [001]_h_ in the hexagonal setting; Fig. 1). Let us define [111]_c_ = [001]_h_ as the *z*-direction. On the other hand, the incommensurate cycloidal spin structure with a periodicity of 620 Å suggests the appearance of a small improper polarization in the *R3c* BFO *via* the reverse Dzyloshinskii-Moriya (DM) coupling^21–23^. The spin-density wave (SDW) associated with this incommensurate spin cycloid is characterized by the propagation vector ***Q*** along the [110]_h_ direction in the hexagonal setting^18,19,24,25^. Let us define [110]_h_ as the *x*-direction (Fig. 1). We will first examine the magnitude of this reverse DM coupling-induced parasitic polarization which is directly linked to the spin cycloid before presenting the relevant thermodynamic potential of the bulk *R3c* BFO. The induced polarization (Δ***P***_*DM*_) by the reverse DM interaction is expressed by the following form:

1 $${\rm{\Delta }}{{\boldsymbol{P}}}_{DM}={d}_{DM}{\hat{{\boldsymbol{e}}}}_{ij}\times ({{\boldsymbol{M}}}_{{\boldsymbol{i}}}\times {{\boldsymbol{M}}}_{{\boldsymbol{j}}})$$

where ${\hat{{\boldsymbol{e}}}}_{{ij}}$ denotes a unit vector connecting the two neighboring magnetic (spin) moments, ***M***_***i***_ and ***M***_*j*_, at the sites *i* and *j*, respectively. Thus, we have to evaluate the position-dependent $({{\boldsymbol{M}}}_{{\boldsymbol{i}}}\times {{\boldsymbol{M}}}_{{\boldsymbol{j}}})$ to assess Δ***P***_***DM***_ due to the cycloidal variation of ***M*** with the propagation vector ***Q*** along [110]_h_.

**Figure 2:**
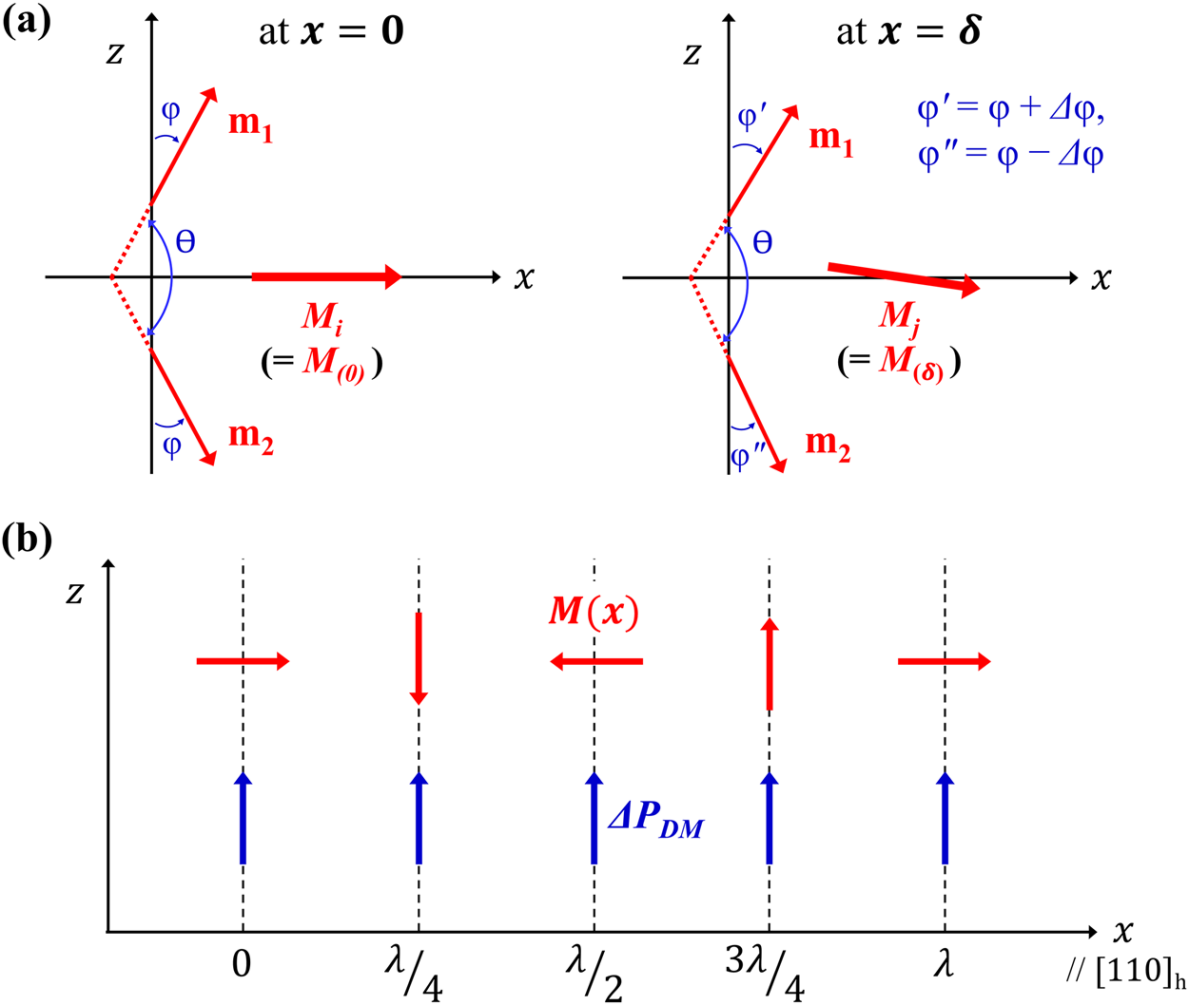
Canted sublattice magnetization vectors and associated polarization in the *R3c* BFO. (**a**) Two sublattice magnetization vectors, ***m***_**1**_ and ***m***_**2**_, at *x* = 0 (left-hand side) and at *x* = *δ* (right-hand side), projected on the hexagonal *x*-*z* plane. The figure shows *Δφ*-degree clockwise rotation of ***m***_**1**_ or ***m***_**2**_, as the cycloidal spin-density wave (SDW) proceeds from *x* = 0 to *x* = *δ* along the [110]_h_ SDW propagation axis. (**b**) Cycloidal variation of ***M***(***x***) along [110]_h_. In contrast, the improper polarization caused by the reverse DM interaction, ${\rm{\Delta }}{{\boldsymbol{P}}}_{DM}$, does uniformly polarize along the *z*-axis and is *x*-location-independent. Here, the *x*-axis is parallel to [110]_h_ and the polar *z*-axis is parallel to [001]_h_ or equivalently to [111]_c_. Thus, the *y*-axis is parallel to [$1\bar{1}0$]_*h*_.

Let us call the net magnetic moment at *x* = 0 as ***M***_***i***_ (i.e., the site *i* at *x* = 0). Then, ***M***_***i***_ is given by the sum of the two neighboring canted sublattice magnetization vectors, ***m***_**1**_ and ***m***_**2**_, along the *z*-direction (but at the same *x* = 0). Since the two neighboring canted magnetizations exhibit a canted antiferromagnetic (AFM) coupling along the *c*-axis (i.e., along [001]_h_) with the canting angle $\phi$, ***m***_**1**_ and ***m***_**2**_ can explicitly be written as ${{\boldsymbol{m}}}_{{\boldsymbol{1}}}={{m}}_{{o}}$ $(\cos \,{\phi }\,\,\hat{{\boldsymbol{z}}}+\,\sin \,{\phi }\,\hat{{\boldsymbol{x}}})$ $+{m}_{y}\hat{{\boldsymbol{y}}}\,$  and   ${{\boldsymbol{m}}}_{{\bf{2}}}={m}_{o}(-\cos \,\phi \,\hat{{\boldsymbol{z}}}\,+\,\sin \,\phi \hat{{\boldsymbol{x}}})+{m}_{y}\hat{{\boldsymbol{y}}}$, where *m*_*o*_ denotes the magnitude of ***m***_**1**_ (or ***m***_**2**_) vector projected on the *x*-*z* plane (Fig. 2) and ${m}_{y}$ designates the *y*-component of canted spin moment with its magnitude given by *m*_*y*_ = *m*_*o*_sin*χ* = *m*_*o*_sin(0.203°) = 0.0035*m*_*o*_^17^. Thus, ***M***_***i***_ (≡***M*** $(0)$) is given by

2 $${{\boldsymbol{M}}}_{{\boldsymbol{i}}}=2{{m}}_{{o}}\,\sin \,{\phi }\,\hat{{\boldsymbol{x}}}+2{m}_{y}\hat{{\boldsymbol{y}}}$$

On the other hand, the two neighboring canted magnetizations, ***m***_**1**_ and ***m***_**2**_, at the site *j*, that are $\delta$ away from *x* = 0 are given by ${{\boldsymbol{m}}}_{1}={m}_{o}(\cos \,\phi ^{\prime} \hat{{\boldsymbol{z}}}+\,\sin \,\phi ^{\prime} \hat{{\boldsymbol{x}}})+{m}_{y}\hat{{\boldsymbol{y}}}$ and ${{\boldsymbol{m}}}_{2}={m}_{o}(-\cos \,\phi ^{\prime\prime} \hat{{\boldsymbol{z}}}+\,\sin \,\phi ^{\prime\prime} \hat{{\boldsymbol{x}}})+{m}_{y}\hat{{\boldsymbol{y}}}$, where $\phi ^{\prime} \equiv \phi +{\rm{\Delta }}\phi$ and $\phi ^{\prime\prime} \equiv \phi -{\rm{\Delta }}\phi$ with the variation of the spin-canting angle (*Δφ*) associated with the translation from *x* = 0 to *x* = $\delta$ along [110]_h_. Thus, ${{\boldsymbol{M}}}_{{\boldsymbol{j}}}[\equiv {\boldsymbol{M}}(\delta )]$ is given by ${{\boldsymbol{M}}}_{{\boldsymbol{j}}}={m}_{o}\{\sin \phi ^{\prime} +\,\sin \,\phi ^{\prime\prime} \}\,\hat{{\boldsymbol{x}}}+{m}_{o}\{\cos \phi ^{\prime} -\,\cos \,\phi ^{\prime\prime} \}\,\hat{{\boldsymbol{z}}}+2{m}_{y}\hat{{\boldsymbol{y}}}$. This expression is readily transformed into the following form using elementary trigonometric algebra:

3 $${{\boldsymbol{M}}}_{{\boldsymbol{j}}}=2{m}_{o}\,\sin \,\phi \,\cos ({\rm{\Delta }}\phi )\hat{{\boldsymbol{x}}}-2{m}_{o}\,\sin \,\phi \,\sin ({\rm{\Delta }}\phi )\hat{{\boldsymbol{z}}}+2{m}_{y}\hat{{\boldsymbol{y}}}$$

Plugging Eqs (2) and (3) into Eq. (1) yields the following expression for the improper polarization induced by the reverse DM coupling:

4 $$\begin{array}{rcl}{\rm{\Delta }}{{\boldsymbol{P}}}_{DM} & = & 4{m}_{o}^{2}{d}_{DM}{\sin }^{2}\phi \,\sin ({\rm{\Delta }}\phi )\hat{{\boldsymbol{z}}}-4{m}_{o}{m}_{y}\,\sin \,\phi \{1-\,\cos ({\rm{\Delta }}\phi )\}\hat{{\boldsymbol{y}}}\\  & = & {m}_{o}^{2}\,\sin \,\phi [4{d}_{DM}\,\sin \,\phi \,\sin \,({\rm{\Delta }}\phi )\hat{{\boldsymbol{z}}}-0.014\{1-\,\cos ({\rm{\Delta }}\phi )\}\hat{{\boldsymbol{y}}}]\\  & \approx  & 4{m}_{o}^{2}{d}_{DM}\,{\sin }^{2}\phi \,\sin \,({\rm{\Delta }}\phi )\hat{{\boldsymbol{z}}}\end{array}$$

The last expression of ${{\boldsymbol{P}}}_{DM}$ is valid if $\cos ({\rm{\Delta }}\phi )\approx 1$. As defined previously, ${\rm{\Delta }}\phi$ denotes the variation of the spin-canting angle associated with the translation from one Fe-site to the nearest-neighbor Fe-site along the *x*-axis. Thus, Δ*φ* = 360° $\times$ (5.58 Å/620 Å) = 3.24°, where 5.58 Å does correspond to the distance between the two nearest-neighbor Fe ions along the *x*-axis^17^. This predicts that $\cos ({\rm{\Delta }}\phi )=0.9984\approx 1$ and supports the validity of the last expression in Eq. (4). We will quantitatively examine the validity of this proposition of ignoring the *y*-component of the reverse DM coupling induced polarization in ‘Discussion’ section. It is interesting to notice that ${{\boldsymbol{P}}}_{DM}$ is location-independent but depends on the DM coupling strength (*d*_*DM*_), canting angle $(\phi ),$ and the periodicity of SDW through ${\rm{\Delta }}\phi$. Thus, the improper polarization induced by the reverse DM coupling does uniformly polarizes along the *z*-axis, i.e., along [001]_h_ [Fig. 2].

Within a continuum approximation for magnetic properties, the DM interaction responsible for this cycloidal modulation of spin moments in the *R3c* BFO can be expressed by inhomogeneous invariants, so-called ‘Lifshitz invariants’ in the free-energy density^26^. Since the leading terms in the DM interaction are linear with respect to first spatial derivatives of magnetization in an antisymmetric mathematical form, the Lifshitz invariant for the *R3c* BFO can be written as

5 $$\begin{array}{ccc}{\rm{\Delta }}{f}_{LF} & = & {\gamma }_{s}{P}_{z}({L}_{z}\frac{{\rm{\partial }}{L}_{x}}{{\rm{\partial }}x}-{L}_{x}\frac{{\rm{\partial }}{L}_{z}}{{\rm{\partial }}x})+{\gamma {}^{{\rm{^{\prime} }}}}_{s}{P}_{x}({L}_{x}\frac{{\rm{\partial }}{L}_{z}}{{\rm{\partial }}z}-{L}_{z}\frac{{\rm{\partial }}{L}_{x}}{{\rm{\partial }}z})\\  &  & +\frac{1}{2}{\kappa }_{s}\{{(\frac{{\rm{\partial }}{L}_{x}}{{\rm{\partial }}x})}^{2}+{(\frac{{\rm{\partial }}{L}_{z}}{{\rm{\partial }}x})}^{2}+{(\frac{{\rm{\partial }}{L}_{x}}{{\rm{\partial }}z})}^{2}+{(\frac{{\rm{\partial }}{L}_{z}}{{\rm{\partial }}z})}^{2}\}\end{array}$$

where ${\gamma }_{s}$ and ${\gamma ^{\prime} }_{s}$ denote the Lifshitz relativistic *P*-*L* exchange-coupling constants. Since the *R3c* BFO is antiferromagnetic (AFM), we used the magnitude of the AFM Néel vector, *L*, instead of the net magnetization order parameter, *M*, [$\equiv |{{\boldsymbol{m}}}_{{\bf{1}}}+{{\boldsymbol{m}}}_{{\bf{2}}}|]$, in the description of ${\rm{\Delta }}{f}_{LF}$. Herein, the DM vector is replaced by ${\gamma }_{s}P$ since the polarization couples to gradients of *M* or *L*, thereby inducing an inhomogeneous cycloidal spin configuration. In the next section, we will use this form of ${\rm{\Delta }}{f}_{LF}$ in constructing the thermodynamic potential for the *R3c* BFO.

### Landau-Lifshitz-Ginzburg Thermodynamic Potential

Before presenting the free-energy density of the multiferroic *R3c* BiFeO_3_ (BFO), we have first examined the free-energy density of the ferroelectric subsystem, ${\rm{\Delta }}f(P),$ on the basis of Landau-Ginzburg-Devonshire phenomenological theory^27–29^. As mentioned previously, the *R3c* BFO is represented by a pseudo-cubic unit cell with its proper polarization along the cubic [111]_c_ direction (i.e., [001]_h_ in the hexagonal setting; Fig. 1). Then, the free-energy density (thermodynamic potential) of the ferroelectric subsystem can be expanded on the basis of a paraelectric prototypic cell having cubic ${P}_{m\bar{3}m}$ symmetry:

6 $${\rm{\Delta }}f(P)={f}_{o}+\frac{1}{2}{\chi }_{p}({P}_{x}^{2}+{P}_{y}^{2}+{P}_{z}^{2})+{\xi ^{\prime} }_{p}({P}_{x}^{4}+{P}_{y}^{4}+{P}_{z}^{4})+{\xi ^{\prime\prime} }_{p}({P}_{x}^{2}{P}_{y}^{2}+{P}_{y}^{2}{P}_{z}^{2}+{P}_{z}^{2}{P}_{x}^{2})$$

where *χ*_*p*_ denotes the dielectric stiffness, and ${\xi ^{\prime} }_{p},\,{\xi ^{\prime\prime} }_{p}$ are high-order stiffness coefficients. In Eq. (6), the three polarization components along the three orthogonal cubic directions are denoted by *P*_*x*_,*P*_*y*_, and *P*_*z*_. Then, the proper ferroelectric polarization (*P*) along the pseudo-cubic [111]_c_ is given by ${P}^{2}={P}_{x}^{2}+{P}_{y}^{2}+{P}_{z}^{2}$. Substituting this relation into Eq. (6) and suitably rearranging the resulting relation, one can obtain the following relation:

7 $${\rm{\Delta }}f(P)={f}_{o}+\frac{1}{2}{\chi }_{p}{P}^{2}+{\xi ^{\prime} }_{p}{P}^{4}+{\xi \prime\prime\prime }_{p}({P}_{x}^{2}{P}_{y}^{2}+{P}_{y}^{2}{P}_{z}^{2}+{P}_{z}^{2}{P}_{x}^{2})$$

where ${\xi \prime\prime\prime }_{p}$ is defined by ${\xi \prime\prime\prime }_{p}\equiv {\xi ^{\prime\prime} }_{p}-2{\xi ^{\prime} }_{p}.$ Since *P* is parallel to [111]_c_, ${P}_{x}={P}_{y}={P}_{z}=\tfrac{1}{\sqrt{3}}P$. Substituting this relation into Eq. (7), one can immediately obtain the following expression:

8 $${\rm{\Delta }}f(P)={f}_{o}+\frac{1}{2}{\chi }_{p}{P}^{2}+\frac{1}{4}{\xi }_{p}{P}^{4}$$

where *ξ*_*p*_ is defined by ${\xi }_{p}\equiv \frac{4}{3}(3{\xi ^{\prime} }_{p}+{\xi \prime\prime\prime }_{p}) > 0.$

**Figure 3:**
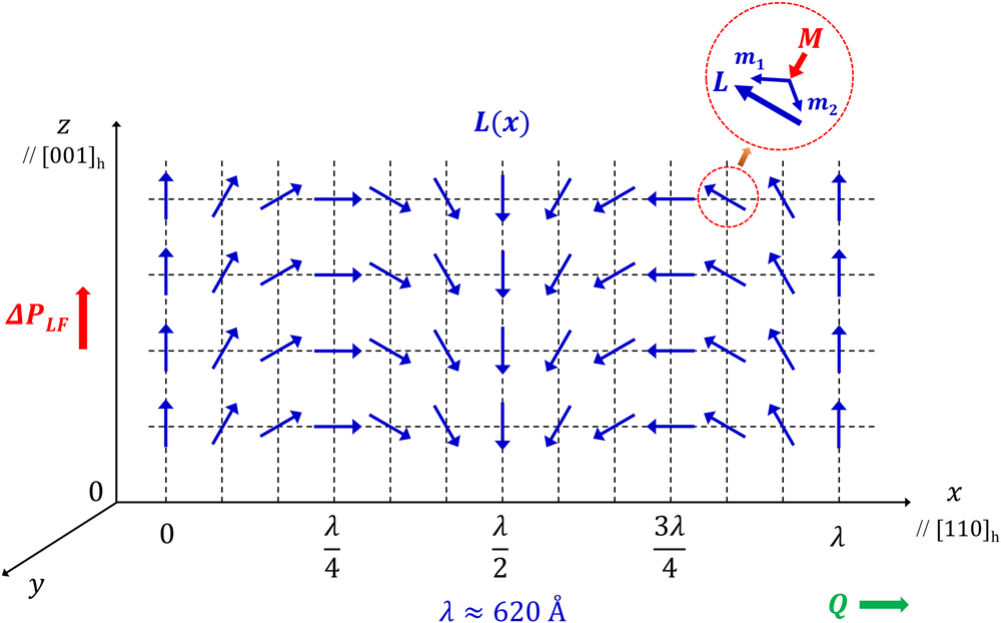
A two dimensional representation of the AFM Néel vector, ***L***(***x***), that forms a continuously varying SDW with the propagation vector ***Q*** along the [110]_h_. Herein, the improper polarization arising from the Lifshitz exchange coupling in the SDW is denoted by ${\rm{\Delta }}{{\boldsymbol{P}}}_{LF}$ and is predicted to be parallel to the *z*-axis or [001]_h_.

Considering the Lifshitz invariant for the cycloidal modulation of spin moments [Eq. (5)] and the free-energy density for the ferroelectric subsystem [Eq. (8)], one can write down the Landau-Lifshitz-Ginzburg thermodynamic potential of the single crystalline *R3c* BFO in terms of two independent order parameters, *P* and *L*, where *L* is an AFM Néel vector describing the staggered sublattice magnetization. The model free-energy density $({\rm{\Delta }}f)$ with respect to the paraphrase where $\langle P\rangle =\langle L\rangle =0$ is

9 $$\begin{array}{ccc}{\rm{\Delta }}f(P,\,L) & = & \frac{1}{2}{\chi }_{p}{P}^{2}+\frac{1}{4}{\xi }_{p}{P}^{4}+\frac{1}{2}{\chi }_{l}{L}^{2}+\frac{1}{4}{\xi }_{l}{L}^{4}-{\gamma }_{q}{P}^{2}({{\boldsymbol{m}}}_{{\bf{1}}}\cdot {{\boldsymbol{m}}}_{{\bf{2}}})+\frac{1}{2}{\kappa }_{G}\sum _{i=x,y,z}({\boldsymbol{\nabla }}{L}_{i}{)}^{2}\\  &  & +\,{\gamma }_{s}{P}_{z}({L}_{z}\frac{{\rm{\partial }}{L}_{x}}{{\rm{\partial }}x}-{L}_{x}\frac{{\rm{\partial }}{L}_{z}}{{\rm{\partial }}x})+{\gamma }_{s}{\rm{^{\prime} }}{P}_{x}({L}_{x}\frac{{\rm{\partial }}{L}_{z}}{{\rm{\partial }}z}-{L}_{z}\frac{{\rm{\partial }}{L}_{x}}{{\rm{\partial }}z})\\  &  & +\,\frac{1}{2}{\kappa }_{s}\{{(\frac{{\rm{\partial }}{L}_{x}}{{\rm{\partial }}x})}^{2}+{(\frac{{\rm{\partial }}{L}_{z}}{{\rm{\partial }}x})}^{2}+{(\frac{{\rm{\partial }}{L}_{x}}{{\rm{\partial }}z})}^{2}+{(\frac{{\rm{\partial }}{L}_{z}}{{\rm{\partial }}z})}^{2}\}\end{array}$$

where *P* denotes the magnitude of the total ferroelectric polarization (proper + improper) developed along the hexagonal *c*-axis, *i*.*e*., [001]_h_, or, equivalently, along [111]_c_ of the pseudo-cubic unit cell (Fig. 1)^13,14^. According to our Berry-phase calculations, *P* ≈ *P*_*z*_ and *P*_*z*_ is as high as ~90 *μ*C/cm^2^ for the undoped BFO having the *R3c* space-group symmetry^17^, where *P*_*z*_ designates the proper off-centering polarization developed along the hexagonal [001] direction. Here, we would like to remind that ‘*z*’ does not refer to the cubic [001] direction. In addition, *P*_*x*_ = 0 as the SDW-propagation direction (***Q***) is parallel to *x* [Fig. 3]. Equation (4) formally supports this conclusion. Several previous investigators adopted similar forms of the free-energy density in their theoretical analysis of the *R3c* BFO^24,30–32^. However, the present form [Eq. (9)] is best suited to theoretical treatment of the spin-coupling-induced improper polarizations and the latent magnetization that are the two main subjects of the present study. The magnitude of the AFM Néel vector, *L*, is defined as *L* = |***m***_**1**_ − ***m***_**2**_|, where ***m***_**1**_ and ***m***_**2**_ denote two canted neighboring sublattice magnetization vectors^24^. Then, it can be shown immediately that *M* and *L* are not independent of each other but are interrelated by *M* ^2^ − *L* ^2^ = 4(***m***_**1**_ · ***m***_**2**_). Thus, the magnetostrictive coupling invariant in Eq. (9) can be rewritten using the following biquadratic form:

$${\rm{\Delta }}{f}_{ms}\,(\equiv {\rm{\Delta }}\,{f}_{q})=-{\gamma }_{q}{P}^{2}({{\boldsymbol{m}}}_{{\bf{1}}}\cdot {{\boldsymbol{m}}}_{{\bf{2}}})=-\frac{1}{4}{\gamma }_{q}{P}^{2}({M}^{2}-{L}^{2}).$$

### Lifshitz Invariant associated with Cycloidal Spin-density Wave

As described previously, the single crystalline *R3c* BFO is characterized by the incommensurate SDW with the propagation vector ***Q*** along the [110]_h_ spiral direction in hexagonal setting^18,19,24^. As schematically shown in Fig. 3, the *x*-location-dependent Néel vector [***L***(*x*)] forms a continuously varying cycloidal vector on the *x*-*z* plane with its propagation direction ***Q*** along $\hat{{\boldsymbol{x}}}$ (=[110]_h_). Thus, the Néel vector is given by ${\boldsymbol{L}}(x)={L}_{x}\hat{{\boldsymbol{x}}}+{L}_{z}\hat{{\boldsymbol{z}}}$. Since the Néel vector ***L***(*x*) lies on the *x*-*z* plane, *L*_*y*_ **=** 0. Accordingly, we establish the following expressions for two orthogonal components of ***L***(*x*):

10 $${L}_{x}={L}_{o}\,\sin (Qx)\,\,{\rm{a}}{\rm{n}}{\rm{d}}\,\,{L}_{z}={L}_{o}\,\cos (Qx)$$

where *Q* ≡ |***Q***| = 2*π*/*λ* with *λ* = 620 Å. One can readily obtain the following relations for *M*_*x*_ and *M*_*z*_ from Eq. (10): *M*_*x*_ = *M*_*o*_cos(*Qx*) and *M*_*z*_ = −*M*_*o*_sin(*Qx*). Thus, ***M***(*x*) and ***L***(*x*) vectors are perpendicular to each other.

Let us then find a Lifshitz invariant arising from this cycloidal SDW. One can compactly rewrite the Lifshitz invariant by assuming that ${\gamma }_{s}={\gamma }_{s}^{\prime}$ for simplicity.

11 $$\begin{array}{rcl}{\rm{\Delta }}{{f}}_{{LF}} & = & {{\gamma }}_{{s}}{\boldsymbol{P}}\cdot \{{\boldsymbol{L}}({\boldsymbol{\nabla }}\cdot {\boldsymbol{L}})-({\boldsymbol{L}}\cdot {\boldsymbol{\nabla }}){\boldsymbol{L}}\}+\frac{{1}}{{2}}{{k}}_{{s}}\{{({\nabla }_{{x}}{{L}}_{{x}})}^{2}+{({\nabla }_{{x}}{{L}}_{{z}})}^{2}+{({\nabla }_{{z}}{{L}}_{{x}})}^{2}+{({\nabla }_{{z}}{{L}}_{{z}})}^{2}\}\\  & \approx  & {{\gamma }}_{{s}}{\boldsymbol{P}}\cdot \{{\boldsymbol{L}}({\boldsymbol{\nabla }}\cdot {\boldsymbol{L}})-({\boldsymbol{L}}\cdot {\boldsymbol{\nabla }}){\boldsymbol{L}}\}\end{array}$$

In obtaining the last expression, we omitted terms representing the square of Néel-vector gradients. According to Mostovoy^33^, these nonlinear square terms do not practically contribute to the uniform improper polarization caused by the Lifshitz *P*-*L* exchange coupling. Since ${\boldsymbol{L}}(x)={L}_{x}\hat{{\boldsymbol{x}}}+{L}_{z}\hat{{\boldsymbol{z}}}$, it can be shown that the Lifshitz invariant is given by

12 $$\begin{array}{rcl}{\rm{\Delta }}{{f}}_{{LF}} & \approx  & {{\gamma }}_{s}\{{P}_{x}\hat{{\boldsymbol{x}}}+{P}_{y}\hat{{\boldsymbol{y}}}+{P}_{z}\hat{{\boldsymbol{z}}}\}\cdot \{({L}_{x}\frac{\partial {L}_{z}}{\partial z}-{L}_{z}\frac{\partial {L}_{x}}{\partial z})\hat{{\boldsymbol{x}}}+({L}_{z}\frac{\partial {L}_{x}}{\partial x}-{L}_{x}\frac{\partial {L}_{z}}{\partial x})\hat{{\boldsymbol{z}}}\}\\  & = & {{\gamma }}_{s}{P}_{z}({L}_{z}\frac{\partial {L}_{x}}{\partial x}-{L}_{x}\frac{\partial {L}_{z}}{\partial x})\end{array}$$

In obtaining the last expression, we used the equality that $\frac{{\rm{\partial }}{L}_{z}}{{\rm{\partial }}z}=\frac{{\rm{\partial }}{L}_{x}}{{\rm{\partial }}z}=0$ since both *L*_*x*_ and *L*_*z*_ are independent of the *z*-coordinate [Fig. 3] and that *P*_*x*_ = 0 as the SDW-propagation direction (***Q***) is parallel to *x*. Combining this result with the two relations given in Eq. (10), one can obtain the following expression of the Lifshitz invariant:

13 $${\rm{\Delta }}{f}_{LF}={\gamma }_{s}{P}_{z}{L}_{o}^{2\,}\{{\cos }^{2}(Qx)+{\sin }^{2}(Qx)\}Q=\,{\gamma }_{s}{P}_{z}{L}_{o}^{2\,}Q$$

where *L*_*o*_(≡ *L* = |***m***_**1**_ − ***m***_**2**_|) denotes the magnitude of Néel vector and is given by ${L}_{o}^{2}=4{m}_{o}^{2}{\cos }^{2}\phi \approx 4{m}_{o}^{2}$ since *φ* ≈ 0.7° [Fig. 2].

R. de Sousa and J. E. Moore^24^ used another form of the Lifshitz exchange coupling for the *R3c* BFO, which is apparently different from Δ*f*_*LF*_ presented in Eq. (11). It is given by

14 $${\rm{\Delta }}{f^{\prime} }_{LF}={\gamma }_{s}{\boldsymbol{P}}\cdot \{{\boldsymbol{L}}({\boldsymbol{\nabla }}\cdot {\boldsymbol{L}})+{\boldsymbol{L}}\times ({\boldsymbol{\nabla }}\times {\boldsymbol{L}})\}$$

After carrying out several steps for mathematical rearrangements, one can show that

15 $${\rm{\Delta }}{f^{\prime} }_{LF}={\gamma }_{s}[\{{P}_{x}{L}_{x}(\frac{\partial {L}_{x}}{\partial x})+{P}_{x}{L}_{z}(\frac{\partial {L}_{z}}{\partial x})\}+\{{P}_{z}{L}_{z}(\frac{\partial {L}_{x}}{\partial x})-{P}_{z}{L}_{x}(\frac{\partial {L}_{z}}{\partial x})\}]$$

In obtaining the above relation, we used the equality that $(\frac{\partial {L}_{x}}{\partial y})=(\frac{\partial {L}_{x}}{\partial z})=(\frac{\partial {L}_{z}}{\partial y})=0$. As mentioned previously, *P*_*x*_ = 0 since the SDW-propagation direction (***Q***) is parallel to *x*. Then, combining Eq. (15) with Eq. (10) immediately yields

16 $${\rm{\Delta }}{f^{\prime} }_{LF}=\,{\gamma }_{s}{P}_{z}({L}_{z}\frac{\partial {L}_{x}}{\partial x}-{L}_{x}\frac{\partial {L}_{z}}{\partial x})={\gamma }_{s}{P}_{z}{L}_{o}^{2\,}Q$$

Accordingly, ${\rm{\Delta }}{f}_{LF}={\rm{\Delta }}{f^{\prime} }_{LF}$. This indicates that the two apparently different forms of the Lifshitz invariant [i.e., Eqs (11) and (14)] are equal to each other under the condition of cycloidal spin ordering confined in an *x*-*z* plane with the translational symmetry along $\hat{{\boldsymbol{y}}}$ and the propagation direction along $\hat{{\boldsymbol{x}}}$ which is parallel to [110]_h_.

### Free-energy Minimization for Deducing Two Distinct Improper Polarizations

Incorporating the results of Eq. (13) for the Lifshitz invariant into Eq. (9), we obtain a simplified form of the thermodynamic potential.

17 $$\begin{array}{c}{\rm{\Delta }}f(P,L)=\,\frac{1}{2}{\chi }_{p}{P}^{2}+\frac{1}{4}{\xi }_{p}{P}^{4}+\frac{1}{2}{\chi }_{l}{L}^{2}+\frac{1}{4}{\xi }_{l}{L}^{4}-\frac{1}{4}{\gamma }_{q}{P}^{2}({M}^{2}-{L}^{2})\\ \quad \quad \quad \quad \quad +\frac{1}{2}{\kappa }_{G}\sum _{i=x,y,z}{({\boldsymbol{\nabla }}{L}_{i})}^{2}+{\gamma }_{s}P{L}^{2}Q\end{array}$$

where we used the notation *P* for the proper off-centering polarization, *P*_*z*_, since *P*_*z*_ ≈ *P*. Then, consider the free-energy functional for a finite volume, Δ*F* = ∫*dv*Δ*f*(*P*,*L*). We impose the following equality for equilibrium: $\delta \Delta F=\int dv\{{(\frac{\partial {\rm{\Delta }}f}{\partial P})}_{L}\delta P+{(\frac{\partial {\rm{\Delta }}f}{\partial L})}_{P}\delta L\}=0$. Since the *P*-*L* cross-coupling is sufficiently weak, one can establish:

18 $$\int dv\,\delta P{(\frac{\partial {\rm{\Delta }}f}{\partial P})}_{L}=\,\int dv\,\delta L{(\frac{\partial {\rm{\Delta }}f}{\partial L})}_{P}=0\,$$

Let us first consider the equilibrium off-centering (proper) polarization. One can immediately establish the following equality by imposing Eq. (18) to Eq. (17):

19 $$\int dv\,\delta P\{\,{\chi }_{p}P+{\xi }_{p}{P}^{3}-\frac{1}{2}{\gamma }_{q}({M}^{2}-{L}^{2})P+{\gamma }_{s}{L}^{2}Q\}=0\,$$

If the Lifshitz-coupling term were absent, one would obtain the following relation from Eq. (19): ${P}_{o}\{{\chi }_{p}+{\xi }_{p}{P}_{o}^{2}-\frac{1}{2}{\gamma }_{q}({M}^{2}-{L}^{2})\}=0.$ Then, one immediately obtains the following expression for the equilibrium polarization (*P*_*o*_) in the absence of the Lifshitz exchange coupling:

20 $${P}_{o}^{2}=-(\frac{1}{{\xi }_{p}})\{{\chi }_{p}-\frac{1}{2}{\gamma }_{q}({M}^{2}-{L}^{2})\}\equiv {({P}_{eq(0)}+{\rm{\Delta }}{P}_{ms})}^{2}$$

where ${P}_{eq(0)}$ denotes the equilibrium (proper) off-centering polarization in the absence of any intrinsic ME coupling and ${\rm{\Delta }}{P}_{ms}$ ($\equiv |{\rm{\Delta }}{{\boldsymbol{P}}}_{ms}|$) represents a small improper polarization caused by the magnetostrictive exchange coupling. Thus, ${P}_{o}$ is comprised of two distinct terms, namely, ${P}_{o}={P}_{eq(0)}+{\rm{\Delta }}{P}_{ms}$. Since ${P}_{eq(0)}\gg {\rm{\Delta }}{P}_{ms},$ we establish the following relations from Eq. (20):

21 $${({P}_{eq(0)})}^{2}=-(\frac{{\chi }_{p}}{{\xi }_{p}})\,\,\,{\rm{a}}{\rm{n}}{\rm{d}}\,\,\,{\rm{\Delta }}{P}_{ms}=\frac{{\gamma }_{q}({M}^{2}-{L}^{2})}{4{\xi }_{p}{P}_{eq(0)}}=\frac{{\gamma }_{q}(\,{{\boldsymbol{m}}}_{{\bf{1}}}\cdot {{\boldsymbol{m}}}_{{\bf{2}}})}{{\xi }_{p}{P}_{eq(0)}} < 0$$

According to the Curie-Weiss law, ${\chi }_{p}=\frac{4\pi }{c}(T-{T}_{c})$, where *T*_*c*_ denotes the ferroelectric Curie temperature (≈1100  K for the *R3c* BFO)^11,34^. Thus, *χ*_*p*_ < 0 below *T*_*c*_. Let the total equilibrium polarization that satisfies Eq. (19) be *P*_*eq*_. We then establish

22 $${P}_{eq}\equiv {P}_{o}+{\rm{\Delta }}{P}_{LF}=({P}_{eq(0)}+{\rm{\Delta }}{P}_{ms})+{\rm{\Delta }}{P}_{LF}$$

In the above equation, ${\rm{\Delta }}{P}_{LF}$ ($\equiv |{\rm{\Delta }}{{\boldsymbol{P}}}_{LF}|$) appears due to the last term in the parenthesis of Eq. (19) and thus represents a small improper polarization arising from the Lifshitz coupling.

Owing to the Lifshitz invariant, one cannot obtain a correct analytic solution of *P*_*eq*_ from Eq. (19). We thus treat ${\rm{\Delta }}{P}_{LF}$ as a small perturbation to *P*_*o*_ and obtain a reasonably accurate solution for ${\rm{\Delta }}{P}_{LF}$. Substituting Eq. (22) into Eq. (19) yields the following equality:

23 $$\begin{array}{l}\{{\chi }_{p}{P}_{o}+{\xi }_{p}{P}_{o}^{3}-\frac{1}{2}{\gamma }_{q}({M}^{2}-{L}^{2}){P}_{o}\}\\ +{\chi }_{p}{\rm{\Delta }}{P}_{LF}+{\xi }_{p}\{3{P}_{o}^{2}{\rm{\Delta }}{P}_{LF}+3{P}_{o}{({\rm{\Delta }}{P}_{LF})}^{2}\}\\ -\frac{1}{2}{\gamma }_{q}({M}^{2}-{L}^{2}){\rm{\Delta }}{P}_{LF}+{\gamma }_{s}{L}_{o}^{2}Q=0\end{array}$$

As discussed previously, the terms inside the first parenthesis are equal to 0. Neglecting the term containing ${({\rm{\Delta }}{P}_{LF})}^{2}$, we eventually derive the following expressions of ${\rm{\Delta }}{P}_{LF}$:

24 $${\rm{\Delta }}{P}_{LF}=\,\frac{-{\gamma }_{s}\,{L}_{o}^{2}\,Q}{\{-2{\chi }_{p}+{\gamma }_{q}({M}^{2}-{L}^{2})\}}=\,\frac{-{\gamma }_{s}\,{L}_{o}^{2}\,Q}{2{\xi }_{p}{P}_{o}^{2}} > 0\,$$

In obtaining the second expression of Eq. (24), we substitute Eq. (20) for ${P}_{o}^{2}.$ On the other hand, we used the following equality in obtaining the last relation:$\,\,{\chi }_{p}+{\xi }_{p}{P}_{o}^{2}-\frac{1}{2}{\gamma }_{q}({M}^{2}-{L}^{2})=0.$ This equality was previously discussed in conjunction with Eq. (19).

On the other hand, the improper polarization caused by the magnetostrictive exchange coupling (${\rm{\Delta }}{P}_{ms}$) is given in Eq. (21). Let us now rewrite ${\rm{\Delta }}{P}_{ms}$ in terms of ${L}_{o}^{2}$ to compare this with ${\rm{\Delta }}{P}_{LF}$. The term, ***m***_**1**_ · ***m***_**2**_, appeared in Eq. (21) can be rewritten in terms of the AFM spin angle $\theta$ as ${{\boldsymbol{m}}}_{{\bf{1}}}\cdot {{\boldsymbol{m}}}_{{\bf{2}}}\approx {m}_{o}^{2}\,\cos \,\theta =-{m}_{o}^{2}|\cos \,\theta |=-{m}_{o}^{2}|\cos (178.6^\circ )| < 0.$ In addition to this, ${P}_{o}={P}_{eq(0)}+{\rm{\Delta }}{P}_{ms}\approx {P}_{eq(0)},$ and $|{\boldsymbol{L}}|\equiv |{{\boldsymbol{m}}}_{{\bf{1}}}-{{\boldsymbol{m}}}_{{\bf{2}}}|={L}_{o}=2{m}_{o}\,\cos \,\phi =2{m}_{o}\,\cos (0.7^\circ )\approx 2{m}_{o},$ where $\theta +2\phi$ = 180° [Fig. 2(a)]. Incorporating these three results into Eq. (21), one immediately obtains

25 $${\rm{\Delta }}{P}_{ms}=\frac{-{\gamma }_{q}{L}_{o}^{2}|\cos \,\theta |}{4{\xi }_{p}{P}_{o}}\approx \frac{-{\gamma }_{q}{m}_{o}^{2}}{{\xi }_{p}{P}_{o}} < 0$$

The above equation demonstrates that the improper polarization caused by the magnetostrictive interaction belongs to ***S***_*i*_ · ***S***_*j*_-type scalar coupling. According to Eq. (25), $|{\rm{\Delta }}{P}_{ms}|$ is inversely proportional to *P*_*o*_. In contrast, $|{\rm{\Delta }}{P}_{LF}|$ is inversely proportional to the square of *P*_*o*_. Since |cos *θ*| ≈ 1, the ratio of these two antiparallel improper polarizations is obtained from Eqs (24) and (25), namely, $\frac{|\Delta {P}_{LF}|}{|\Delta {P}_{ms}|}=\frac{2|{\gamma }_{s}|Q}{{\gamma }_{q}{P}_{o}}=\frac{4\pi |{\gamma }_{s}|}{{\gamma }_{q}{P}_{o}\lambda }.$ Finally, the corresponding coupling invariant (${\rm{\Delta }}{f}_{ms}$) can be obtained by using the relation described previously, ${{\boldsymbol{m}}}_{{\bf{1}}}\cdot {{\boldsymbol{m}}}_{{\bf{2}}}=-{m}_{o}^{2}|\cos \,\theta |$.

26 $$\Delta {f}_{ms}(\equiv {\rm{\Delta }}{f}_{q})=-\frac{1}{4}{\gamma }_{q}{P}^{2}({M}^{2}-{L}^{2})=-{\gamma }_{q}{P}^{2}({{\boldsymbol{m}}}_{{\bf{1}}}\cdot {{\boldsymbol{m}}}_{{\bf{2}}})=+{\gamma }_{q}{P}^{2}{m}_{o}^{2}|\cos \,\theta |$$

where *P* is nearly equal to *P*_*z*_ or *P*_*o*_. The subscript ‘*q*’ appeared in $\Delta {f}_{q}$ emphasizes that the magnetostrictive interaction is represented by a biquadratic coupling of the form, *P* ^2^ *M* ^2^ or *P* ^2^ *L* ^2^. According to *ϕ* ^4^-expansion adopted in Eq. (8) [or Eq. (17)], the Landau coefficient *ξ*_*p*_ should be positive. Thus, Eq. (26) tells us that the biquadratic *P*-*M* cross-coupling thermodynamically stabilizes the *R3c* BFO system if *γ*_*q*_ < 0. On the contrary, the biquadratic magnetostrictive coupling destabilizes the system with a concomitant decrease in *P*_*o*_ (i.e., ${\rm{\Delta }}{P}_{ms}$ < 0) if  *γ*_*q*_ > 0. According to the experimental result reported by S. Lee *et al*.^25^, the latter case (*γ*_*q*_ **>** 0) is applicable to the *R3c* BFO. Our theoretical estimate also supports this conclusion (‘Discussion’ section).

Since ${\rm{\Delta }}{P}_{LF}$ appeared in Eq. (24) is equal to $|{\rm{\Delta }}{{\boldsymbol{P}}}_{DM}|$ that is given in Eq. (4), one can derive the following analytical expression for the reverse DM interaction coefficient (*d*_*DM*_) which is a measure of the strength of the reverse DM coupling:

27 $${d}_{DM}=\frac{\pi |{\gamma }_{s}|}{\lambda {\xi }_{p}\,\sin (\Delta \phi ){\tan }^{2}\phi {P}_{o}^{2}}$$

As expected, *d*_*DM*_ is proportional to the Lifshitz *P*-*L* exchange-coupling constant (|*γ*_*s*_|) but is inversely proportional to *λ* (wavelength of the SDW, 620 Å along the [110]_h_). To estimate *d*_*DM*_, thus, one should first know *γ*_*s*_, *ξ*_*p*_, and *P*_*o*_. We will show all the details in ‘Discussion’ section.

### Ginzburg Gradient Energy and Equilibrium Magnetic Remanence

Let us examine the second relation of Eq. (18) by applying Eq. (17) to this requirement.

28 $$\int dv\,\delta L{(\frac{\partial {\rm{\Delta }}f}{\partial L})}_{P}=\int dv\,\delta L[{\chi }_{l}L+{\xi }_{l}{L}^{3}+\frac{1}{2}{\gamma }_{q}{P}^{2}L+2{\gamma }_{s}PQL+\frac{1}{2}{\kappa }_{G}\frac{\partial \{{\sum }_{i}{(\nabla {{\boldsymbol{L}}}_{i})}^{2}\}}{\partial L}]=0\,$$

where *κ*_*G*_ denotes the Ginzburg gradient-energy coefficient. The last term inside the parenthesis of Eq. (28) can be rewritten as

29 $$\frac{1}{2}{\kappa }_{G}\frac{{\rm{\partial }}\{{\sum }_{i}{({\boldsymbol{\nabla }}{{\boldsymbol{L}}}_{i})}^{2}\}}{{\rm{\partial }}L}=\frac{1}{2}{\kappa }_{G}\frac{{\rm{\partial }}\{{\sum }_{i}{({\boldsymbol{\nabla }}{{\boldsymbol{L}}}_{i})}^{2}\}}{{\rm{\partial }}{L}_{i}}\frac{{\rm{\partial }}{L}_{i}}{{\rm{\partial }}L}=-{\kappa }_{G}\sum _{i}{{\boldsymbol{\nabla }}}^{2}{L}_{i}{(\frac{{\rm{\partial }}{L}_{i}}{{\rm{\partial }}L})}_{{L}_{j}}$$

Then, we obtain the following type Euler-Lagrange equation from Eq. (28):

30 $$L\{{\chi }_{l}+{\xi }_{l}{L}^{2}+\frac{1}{2}{\gamma }_{q}{P}^{2}+2{\gamma }_{s}PQ\}-{\kappa }_{G}\sum _{i}{\nabla }^{2}{L}_{i}{(\frac{\partial {L}_{i}}{\partial L})}_{{L}_{j}}=0$$

The Ginzburg gradient term in the above equation is comprised of three distinct terms, namely,

31 $$-{\kappa }_{G}\sum _{i}{\nabla }^{2}{L}_{i}{(\frac{\partial {L}_{i}}{\partial L})}_{{L}_{j}}=-{\kappa }_{G}\{\,{\nabla }^{2}{L}_{x}{(\frac{\partial {L}_{x}}{\partial L})}_{{L}_{y},{L}_{z}}+{\nabla }^{2}{L}_{y}{(\frac{\partial {L}_{y}}{\partial L})}_{{L}_{x},{L}_{z}}+{\nabla }^{2}{L}_{z}{(\frac{\partial {L}_{z}}{\partial L})}_{{L}_{x},{L}_{y}}\}$$

Since ∇^2^ *L*_*x*_ = ***∇*** · ***∇*** *L*_*x*_ and $\frac{\partial {L}_{x}}{\partial y}=\frac{\partial {L}_{x}}{\partial z}=0$, it is not difficult to show that ∇^2^ *L*_*x*_ = −*L*_*o*_ *Q* ^2^ sin(*Qx*), where *L*_*o*_ denotes the magnitude of the AFM Néel vector. Similarly, it can be shown readily that ∇^2^ *L*_*z*_ = ***∇*** · ***∇*** *L*_*z*_ = −*L*_*o*_ *Q* ^2^cos(*Qx*).  On the contrary, ∇^2^ *L*_*y*_ = ***∇*** · ***∇*** *L*_y_ = 0. In addition, it is straightforward to show ${(\frac{\partial {L}_{x}}{\partial L})}_{{L}_{y},{L}_{z}}=\frac{{L}_{o}}{{L}_{x}}=\frac{1}{\sin (Qx)}$ and ${(\frac{\partial {L}_{z}}{\partial L})}_{{L}_{x},{L}_{y}}=\frac{{L}_{o}}{{L}_{z}}=\frac{1}{\cos (Qx)}$. Putting all these results into Eq. (31) and rearranging yields to the following expression for the Ginzburg gradient term:

32 $$+\frac{1}{2}{\kappa }_{G}\frac{\partial \{{\sum }_{i}{(\nabla {{\boldsymbol{L}}}_{i})}^{2}\}}{\partial L}=-{\kappa }_{G}\sum _{i}{\nabla }^{2}{L}_{i}{(\frac{\partial {L}_{i}}{\partial L})}_{{L}_{j}}=+2{\kappa }_{G}{L}_{o}{Q}^{2}$$

where *L* = *L*_*o*_. Substituting Eq. (32) into Eq. (28) yields the following expression for the equilibrium magnitude of the AFM Néel vector:

33 $${({L}_{eq})}^{2}={({L}_{eq(0)})}^{2}-\frac{\{\,\frac{1}{2}{\gamma }_{q}{P}^{2}+2{\gamma }_{s}PQ+2{\kappa }_{G}{Q}^{2}\,\}}{{\xi }_{l}}$$

where ${({L}_{eq(0)})}^{2}\equiv -{\chi }_{l}/{\xi }_{l}$. Thus, ${L}_{eq(0)}$ denotes the equilibrium magnitude of the Néel vector in the absence of any coupling (*i*.*e*., *γ*_*q*_ = *γ*_*s*_ = *κ*_*G*_ = 0). Then, the equilibrium magnetic remanence (${M}_{eq}$) is related to ${L}_{eq}$ *via* the following relation: ${({M}_{eq})}^{2}={({L}_{eq})}^{2}+4{({{\boldsymbol{m}}}_{{\bf{1}}}\cdot {{\boldsymbol{m}}}_{{\bf{2}}})}_{eq}$. Combining this relation with Eq. (33) yields

34 $${({M}_{eq})}^{2}={({M}_{eq(0)})}^{2}+4\delta {({{\boldsymbol{m}}}_{{\bf{1}}}\cdot {{\boldsymbol{m}}}_{{\bf{2}}})}_{eq}-\frac{\{\frac{1}{2}{\gamma }_{q}{P}^{2}+2{\gamma }_{s}PQ+2{\kappa }_{G}{Q}^{2}\}}{{\xi }_{l}}$$

where $\delta {({{\boldsymbol{m}}}_{{\bf{1}}}\cdot {{\boldsymbol{m}}}_{{\bf{2}}})}_{eq}\equiv {({{\boldsymbol{m}}}_{{\bf{1}}}\cdot {{\boldsymbol{m}}}_{{\bf{2}}})}_{eq}-{({{\boldsymbol{m}}}_{{\bf{1}}}\cdot {{\boldsymbol{m}}}_{{\bf{2}}})}_{eq(0)}$ and *M*_*eq*(0)_ denotes the equilibrium magnetic remanence in the absence of any coupling. Thus, the Lifshitz exchange coupling enhances ${M}_{eq}$ if ${\gamma }_{s} < 0$ which corresponds to thermodynamically favorable Lifshitz coupling [Eq. (13)]. According to our theoretical estimate, *γ*_*s*_ is indeed negative as described in ‘Discussion’ section. In contrast, the Ginzburg gradient term always suppresses *M*_*eq*_ as ${\kappa }_{G} > 0.$

The Ginzburg gradient energy can be computed by considering the space average of the gradient term in Eq. (17), namely, ${\rm{\Delta }}{f}_{G}=\frac{1}{2}{\kappa }_{G}\langle {\sum }_{i}{({\boldsymbol{\nabla }}{L}_{i})}^{2}\rangle$ = $\frac{1}{2}{\kappa }_{G}\langle {({\boldsymbol{\nabla }}{L}_{x})}^{2}+{({\boldsymbol{\nabla }}{L}_{y})}^{2}+{({\boldsymbol{\nabla }}{L}_{z})}^{2}\rangle$. It can be shown readily that ${({\boldsymbol{\nabla }}{L}_{x})}^{2}=({\boldsymbol{\nabla }}{L}_{x})\cdot ({\boldsymbol{\nabla }}{L}_{x})$ $=\,{L}_{o}^{2}{Q}^{2}\,{\cos }^{2}(Qx)$ and ${({\boldsymbol{\nabla }}{L}_{z})}^{2}={L}_{o}^{2}{Q}^{2}\,{\sin }^{2}(Qx).$ On the contrary, (${\boldsymbol{\nabla }}$ *L*_*y*_)^2^ = 0 since *L*_*y*_ = 0. Thus, the space average of the Ginzburg gradient term is

35 $${\rm{\Delta }}{f}_{G}=\frac{1}{2}{\kappa }_{G}\langle \sum _{i}{({\boldsymbol{\nabla }}{L}_{i})}^{2}\rangle =\frac{1}{2}{\kappa }_{G}{L}_{o}^{2}{Q}^{2}$$

Since *κ*_*G*_ > 0, the Ginzburg gradient term always increases the free-energy density.

## Discussion

### Estimate of the Two distinct Improper Polarizations

Having theoretically identified the two spin-coupling-induced improper polarizations in the *R3c* BFO (i.e., ${\rm{\Delta }}{{P}}_{ms},\,{\rm{\Delta }}{{P}}_{LF}$), we now focus on the numerical estimate of these values with the help of *ab initio* density-functional theory (DFT) calculations and experimental measurements. For this, let us first consider the improper polarization caused by the magnetostrictive exchange coupling, Δ*P*_*ms*_. As given in Eq. (20), this induced polarization is defined by ${\rm{\Delta }}{P}_{ms}\equiv {P}_{o}-{P}_{eq(0)}$. Since *P*_*eq*(0)_ denotes the off-centering (normal) ferroelectric polarization along the polar *z*-axis or [001]_h_ under the imposed condition of ${\boldsymbol{M}}={{\boldsymbol{m}}}_{{\bf{1}}}+{{\boldsymbol{m}}}_{{\bf{2}}}=0$, it does correspond to the Berry-phase polarization^35,36^ obtained without imposing any spin structure (i.e., paramagnetic phase). In contrast, ${P}_{o}$ represents the net off-centering ferroelectric polarization that includes the exchange coupling effect. Thus, *P*_*o*_ corresponds to the Berry-phase polarization computed by imposing the canted sublattice spin structure with ${{\boldsymbol{m}}}_{{\bf{1}}}\cdot {{\boldsymbol{m}}}_{{\bf{2}}}={m}_{o}^{2}\,\cos \,\theta$ = ${m}_{o}^{2}\,\cos (\pi -2\phi )={m}_{o}^{2}\,\cos (178.6^\circ )$. Since Δ*P*_*ms*_ belongs to ${{\boldsymbol{S}}}_{i}\cdot {{\boldsymbol{S}}}_{j}$-type scalar-coupling-induced improper polarization, the computed value of *P*_*o*_ by the Berry-phase method should be independent of the inclusion of spin-orbit coupling effect^22^. Our DFT calculations predict that ${\rm{\Delta }}{P}_{ms}\equiv {P}_{o}-{P}_{eq(0)}=-20\,$ *nC*/*cm* ^2^, which indicates ${\gamma }_{q} > 0$ according to Eq. (25).

**Figure 4:**
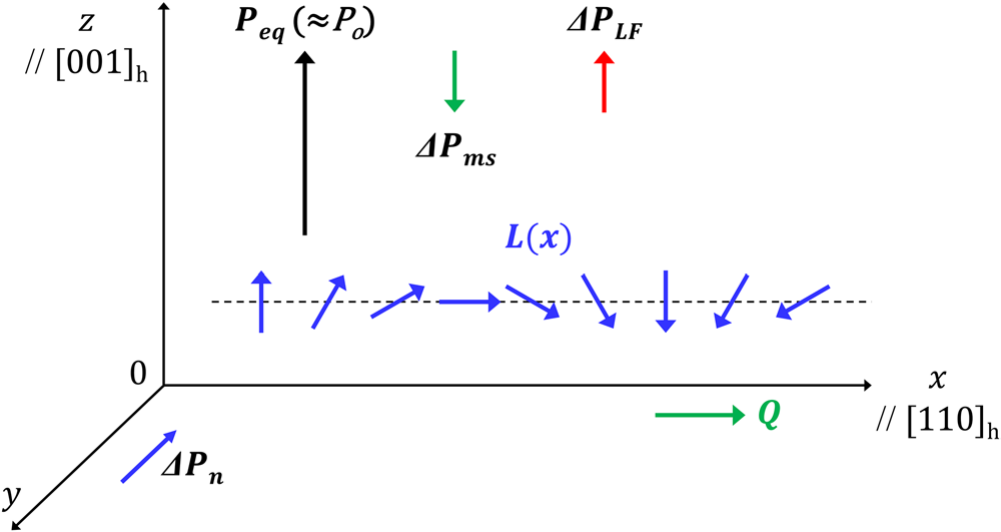
A schematic representation of the off-centering proper ferroelectric polarization, ***P***_*eq*(0)_, and the three parasitic improper polarizations, ${\rm{\Delta }}{{\boldsymbol{P}}}_{ms},\,\,{\rm{\Delta }}{{\boldsymbol{P}}}_{LF},$ and ${\rm{\Delta }}{{\boldsymbol{P}}}_{n}$ in the *R3c* BFO. Among these three improper polarizations, ${\rm{\Delta }}{{\boldsymbol{P}}}_{LF}$ is parallel to ***P***_*eq*(0)_ but ${\rm{\Delta }}{{\boldsymbol{P}}}_{ms}$ is anti-parallel to ***P***_*eq*(0)_. In contrast, ${\rm{\Delta }}{{\boldsymbol{P}}}_{n}$ is perpendicular to ***P***_*eq*(0)_.

Let us estimate the improper polarization (${\rm{\Delta }}{{P}}_{LF}$) arising from the Lifshitz gradient coupling. For this, we have to first consider the slowly varying spin reorientation with the periodicity of 620 Å along [110]_h_ or *x*-axis. The distance between the two neighboring Fe-spin sites along the [110]_h_ SDW propagation axis is 5.580 Å. Thus, the spin-rotation angle (Δ*ϕ*) between the two neighboring Fe sites in the *x*-*z* plane is given by Δ*ϕ* = 360° $\times$ (5.58/620) = 3.24°. We have imposed the spin-orientation structure and performed *ab initio* calculations by adopting a 2 × 2 × 1 supercell. The estimated *ab initio* value of Δ*P*_*LF*_ is ~15 *nC*/*cm* ^2^. However, this computed value is substantially smaller than the experimental value of 36 *nC*/*cm* ^2^ along [001]_h_^36^. Considering reliability of our *ab initio* value, we will adopt this experimental value in the evaluation of the Lifshitz coupling constant, *γ*_*s*_, in the next section. We schematically depict these two distinct improper polarizations with their directions in Fig. 4 and these can be summarized by the following ratio: −Δ*P*_*ms*_ **:**+Δ*P*_*LF*_ = 20:36 = 5:9.

### Theoretical Estimate of the Three Relevant Coupling Constants

**Figure 5:**
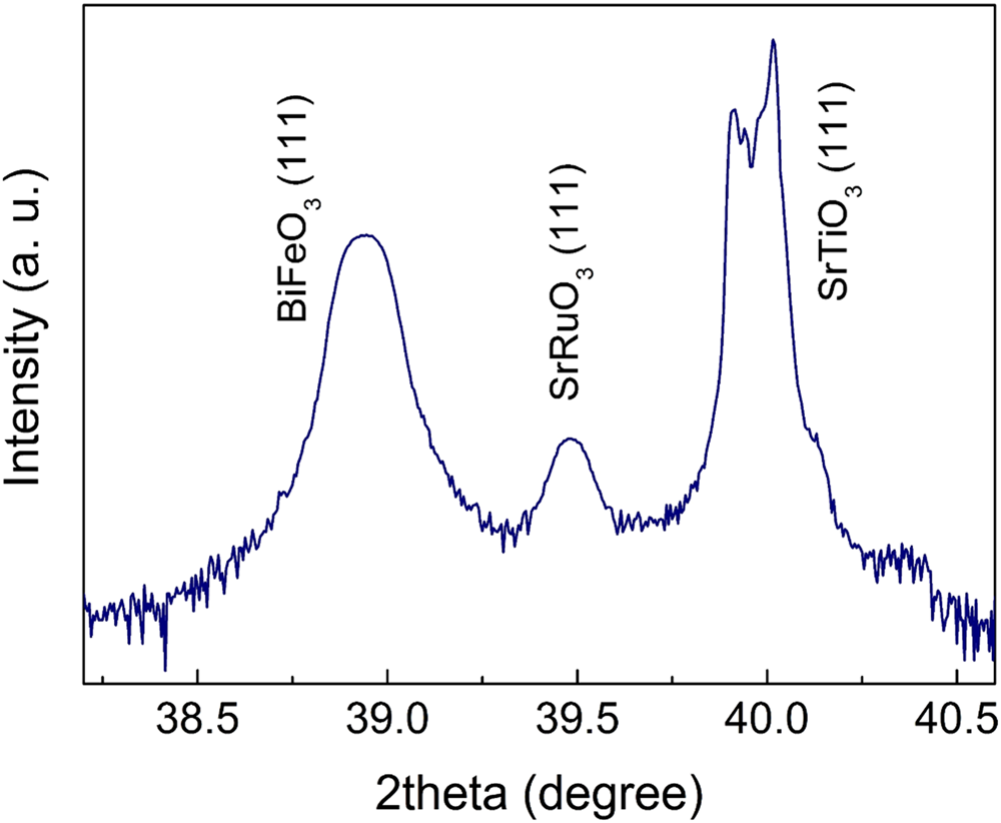
High-resolution theta-2theta x-ray diffraction pattern (XRD) of the BiFeO_3_(BFO)/SrRuO_3_/SrTiO_3_(111) thin-film heterostructure obtained using pulsed laser deposition. As shown, all three layers are characterized by the [111]_c_-preferential growth. The thicknees of the ferroelectric BFO layer grown along the pseudo-cubic [111]_c_ is ~400 nm.

**Figure 6:**
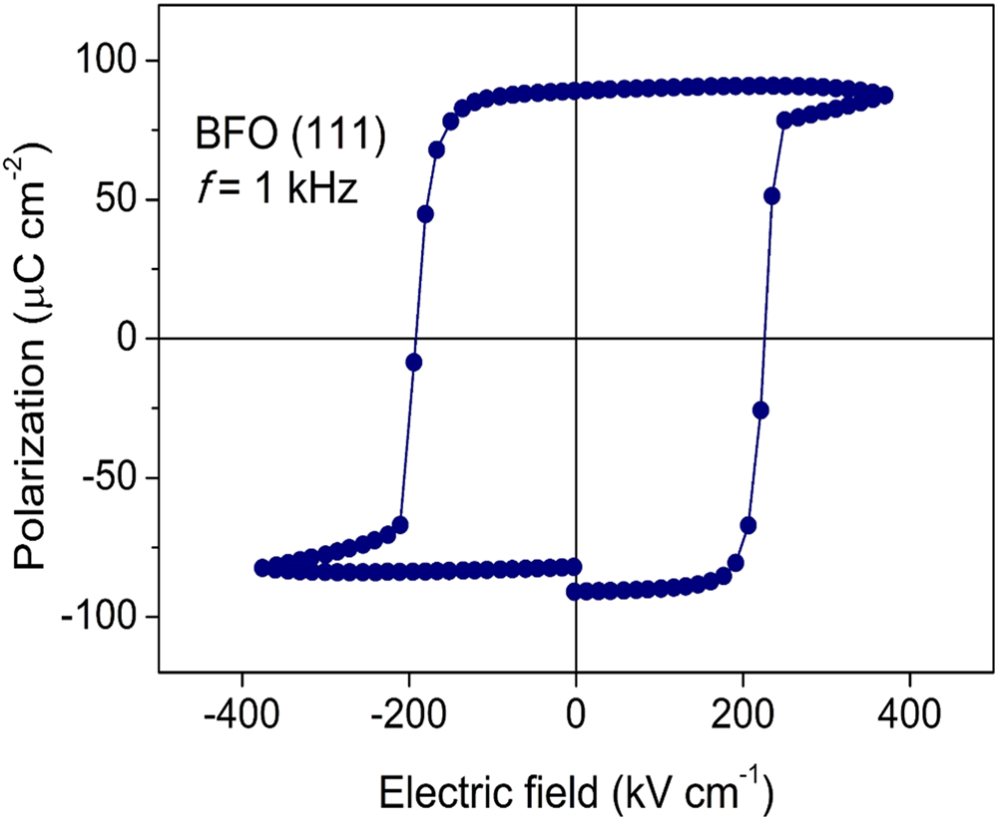
Polarization-electric field (*P−E*) hysteresis loop of the preferential [111]_c_-oriented BFO film (400-nm-thick) obtained at a measuring frequency of 1 kHz at 300 K. As shown, the net switching polarization (2*P*_*r*_) of the [111]_c_-preferentially grown BFO film is ~180 *μC*/*cm* ^2^, which indicates that the remanent polarization (*P*_*r*_) is ~90 *μC*/*cm* ^2^. According to the XRD pattern (Fig. 5), the in-plane film strain in the [111]_c_-oriented BFO layer is fully relaxed at the thickness of 400 nm. Thus, the measured *P*_*r*_ value (~90 *μC*/*cm* ^2^) can be treated as a bulk *P*_*r*_ value of the *R3c* BFO.

Let us first estimate the biquadratic magnetostrictive coupling constant (*γ*_*q*_) by exploiting Eq. (25). As estimated in the previous subsection, $\Delta {P}_{ms}={P}_{o}-{P}_{eq(0)}=-2.0\times {10}^{-4}$ *C*/*m* ^2^. According to our Berry-phase calculations, *P*_*o*_ = 86.3 *μC*/*cm* ^2^ = 0.863 *C*/*m* ^2^. To experimentally check this value, we have fabricated a highly [111]_c_-oriented 400-nm-thick BFO thin film on the (111)SrRuO_3_/SrTiO_3_ substrate [Fig. 5]. As shown in the polarization-electric field (*P*-*E*) hysteresis loop [Fig. 6], the remanent polarization of the [001]_h_-axis-grown BFO film is ~90 *μC*/*cm* ^2^, which nearly coincides with the computed value of *P*_*o*_(=86.3 *μC*/*cm* ^2^).

As can be deduced from Eq. (17), the invariant for the ferroelectric subsystem is given by ${\rm{\Delta }}{f}_{p}=\frac{1}{2}{\chi }_{p}{P}^{2}+\frac{1}{4}{\xi }_{p}{P}^{4}$ **−** ${\gamma }_{q}{P}^{2}({{\boldsymbol{m}}}_{{\bf{1}}}\cdot {{\boldsymbol{m}}}_{{\bf{2}}})\approx \frac{1}{2}{\chi }_{p}{P}^{2}+\frac{1}{4}{\xi }_{p}{P}^{4}$ since the biquadratic exchange coupling contribution is negligibly small as compared with the preceding two terms. From this relation, the Landau *ϕ* ^4^-expansion coefficient *ξ*_*p*_ can be derived in terms of *P*_*eq*_ and ${({\rm{\Delta }}{f}_{p})}_{eq},$ where ${({\rm{\Delta }}{f}_{p})}_{eq}$ denotes the equilibrium free-energy density of the ferroelectric subsystem with respect to that of the paraelectric reference system. This can be written explicitly as

36 $${\xi }_{p}=-4\,{({\rm{\Delta }}{f}_{p})}_{eq}\cdot {({P}_{eq})}^{-4}$$

where (*P*_*eq*_)^2^ = −*χ*_*p*_/*ξ*_*p*_. On the other hand, *P*_*eq*_(=*P*_*o*_) = 0.863 *C*/*m* ^2^. According to the *ab initio* calculations^17^, ${({\rm{\Delta }}{f}_{p})}_{eq}=-0.381\,eV$ per hexagonal cell containing six BFO formula cells (*a* = 5.57987 Å, *c* = 13.87229 Å), which is equivalent to −0.163 × 10^+9^ *J*/*m* ^3^. Plugging these two values into Eq. (36), one finds that ${\xi }_{p}=+1.175\times {10}^{+9}$ $(J\cdot {m}^{5}/{C}^{4}).\,$ On the other hand, ${m}_{o}\approx 5{\mu }_{B}=4.637\times {10}^{-23}\,(J\cdot {T}^{-1})$ for the high-spin Fe^3+^ in the *R3c* BiFeO_3_ (ref.^2^). In addition, $|\cos \,\theta |=|\cos (\pi -2\phi )|=|\cos (178.6^\circ )|\approx 1$. Using all these values and rewriting Eq. (25) in terms of *γ*_*q*_, we obtain

37 $${\gamma }_{q}=\frac{-{\xi }_{p}{P}_{o}\{{P}_{o}-{P}_{eq(o)}\}}{{m}_{o}^{2}}=+9.43\times {10}^{+49}\,(m\cdot {T}^{2}/J\cdot {C}^{2})$$

To estimate the Lifshitz exchange-coupling constant (*γ*_*s*_), we have reconsidered Eq. (24). As mentioned previously, we have adopted the experimental value of 3.6 × 10^−4^ (*C*/*m* ^2^) as Δ*P*_*LF*_. In addition to this, we use the following values for the estimate of *γ*_*s*_: *P*_*o*_ = 0.863 (*C*/*m* ^2^), ${\xi }_{p}=+1.175\times {10}^{+9}(J\cdot {m}^{5}/{C}^{4}),$ *Q* = 2*π*/*λ* = 1.013 × 10^+8^ (*m* ^−1^), and ${L}_{o}^{2}=4{m}_{o}^{2}\,{\cos }^{2}\phi \approx 4{m}_{o}^{2}$ with ${m}_{o}=4.637\times {10}^{-23}\,(J\cdot {T}^{-1})$. Using these five values and rewriting Eq. (24) in terms of *γ*_*s*_, we obtain

38 $${\gamma }_{s}=\frac{-2\,{\xi }_{p}{P}_{o}^{2}{\rm{\Delta }}{P}_{LF}}{{L}_{o}^{2}\,Q}=-7.23\times {10}^{+41}({T}^{2}/J\cdot C)$$

As discussed previously, a negative sign of *γ*_*s*_ indicates that the cycloidal spin ordering is formed spontaneously in the unstrained *R3c* BFO. Let us now estimate *d*_*DM*_, a measure of the reverse DM interaction, using Eq. (27). Then, plugging *P*_*o*_ = 0.863 (*C*/*m* ^2^), ${\xi }_{p}=+1.175\times {10}^{+9}(J\cdot {m}^{5}/{C}^{4}),$ *λ* = 620 Å, Δ*φ* = 3.24°, *φ* = 0.7°, and ${\gamma }_{s}=-7.23\times {10}^{+41}({T}^{2}/J\cdot C)$ into Eq. (27), we obtain ${d}_{DM}=+4.98\times {10}^{+44}({T}^{2}/J\cdot V\cdot {m}^{2})$.

Having estimated *d*_*DM*_, we are now ready to examine our previous proposition that the *y*-component value of the reverse DM coupling-induced polarization [≡Δ*P*_*DM*_(*y*) or ΔP_n_] is relatively negligible as compared with the corresponding *z*-component value [$\equiv {\rm{\Delta }}{P}_{DM}(z)$] (See, the previous section of ‘Improper Polarization and Invariant caused by the Reverse DM Interaction’). According to Eq. (4), the ratio of the two perpendicular improper polarizations can be written as

39 $$\frac{{\rm{\Delta }}{P}_{DM}(y)}{{\rm{\Delta }}{P}_{DM}(z)}=\frac{{m}_{y}\{1-\,\cos ({\rm{\Delta }}\phi )\}}{{m}_{o}{d}_{DM}\,\sin \,\phi \,\sin ({\rm{\Delta }}\phi )}$$

We used the following values to estimate the above ratio: *Δφ* = 3.24°, *φ* = 0.7°, *d*_*DM*_ = +4.98 × 10^+44^ (*T* ^2^/*J* · *V* · *m* ^2^), and $({m}_{y}/{m}_{o})=\,\sin (\chi )$, where *χ* denotes the *y*-component spin-canting angle along the *x*-axis. In contrast, *φ* designates the *x*-component spin-canting angle along the *z*-axis [Fig. 2]. According to our previous *ab initio* calculations^17^, *χ* = 0.203° along the *x*-axis, [110]_h_. Plugging all these values into Eq. (39), one obtains that Δ*P*_*DM*_(*y*)/Δ*P*_*DM*_(*z*) is as small as 1.65 × 10^−47^. This clearly justifies our previous proposition that the *y*-component value of the reverse DM coupling-induced polarization is completely negligible as compared with the corresponding *z*-component value.

### Release of the Latent Magnetization in a Constrained Thin Film

According to the study of Bai and co-workers^37^, the epitaxial film-constraint induces the destruction of a spatially modulated cycloidal spin structure in the bulk *R3c* BFO, releasing a latent AFM component locked within the cycloid. This corresponds to a transition from the incommensurately modulated cycloidal spin state to the homogeneously canted spin state in a constrained film with the onset thickness of ~150 nm^38^. Ryu and co-workers^38^ further showed that the release of a latent AFM magnetization associated with the transition to the homogeneous spin state accompanies with a pronounced increase in the magnetic susceptibility (${\chi }_{m}$) in epitaxially constrained BFO thin films. We will quantitatively account for these observations by using Eq. (34). For an epitaxially constrained thin film having a homogeneous spin structure, we impose that ${\gamma }_{s}=0$ and *κ*_*G*_ = 0 by considering the disappearance of a spatially modulated cycloidal spin structure^39^. For a constrained epitaxial thin film, Eq. (34) thus reads:

$${({M}_{eq(f)})}^{2}={({M}_{eq(0)})}^{2}+4\delta {({{\boldsymbol{m}}}_{{\bf{1}}}\cdot {{\boldsymbol{m}}}_{{\bf{2}}})}_{eq,f}-({\gamma }_{q}{P}^{2}/2{\xi }_{l}),$$

where ${M}_{eq(f)}$ denotes the equilibrium magnetic remanence of the constrained epitaxial film. On the other hand, *M*_*eq*(0)_ denotes the equilibrium magnetic remanence in the absence of any ME coupling. Thus, ${({M}_{eq(0)})}^{2}\equiv -{\chi }_{m}/{\xi }_{m}$. Using this relation and Eq. (34) for ${M}_{eq(b)}$, one can derive the following relation for the latent magnetization released by the transition to the homogeneously canted spin state in a constrained film:

40 $${\rm{\Delta }}{M}_{eq}\equiv {M}_{eq(f)}-{M}_{eq(b)}=\frac{2\{{\gamma }_{s}PQ+{\kappa }_{G}{Q}^{2}\}}{{\xi }_{l}\,\{{M}_{eq(f)}+{M}_{eq(b)}\}}\approx \frac{\{-|{\gamma }_{s}|PQ+{\kappa }_{G}{Q}^{2}\}}{{\xi }_{l}\,{M}_{eq(b)}} > 0$$

where *M*_*eq*(*b*)_ denotes the equilibrium magnetic remanence of the bulk *R3c* BFO and *γ*_*s*_ < 0. In obtaining Eq. (40) we used the following equality:

$$\begin{array}{rcl}\{4\delta {({{\boldsymbol{m}}}_{{\bf{1}}}\cdot {{\boldsymbol{m}}}_{{\bf{2}}})}_{eq,f}-4\delta {({{\boldsymbol{m}}}_{{\bf{1}}}\cdot {{\boldsymbol{m}}}_{{\bf{2}}})}_{eq,b}\} & = & 4\{{({{\boldsymbol{m}}}_{{\bf{1}}}\cdot {{\boldsymbol{m}}}_{{\bf{2}}})}_{eq,f}-{({{\boldsymbol{m}}}_{{\bf{1}}}\cdot {{\boldsymbol{m}}}_{{\bf{2}}})}_{eq,b}\}\\  & = & 4{m}_{o}^{2}\{\,\cos \,{\theta }_{f}-\,\cos \,{\theta }_{b}\}\approx 0,\end{array}$$

where the AFM spin angle of the bulk *R3c* BFO (*θ*_*b*_) is essentially unaffected by the formation of a constrained thin film, namely, *θ*_*f*_ = *θ*_*b*_ = 180° − 2*φ* = 178.6°. The inequality sign in Eq. (40) reflects the observation associated with the transition to the homogeneously canted spin state in a constrained epitaxial film.

One can readily obtain the following inequality by considering the right-hand-side of Eq. (40): ${k}_{G}\ge \frac{|{\gamma }_{s}|P}{Q}$. Thus, the lower limit of the Ginzburg gradient-energy coefficient [(*κ*_*G*_)_*l*.*l*._] can be estimated by using ${({k}_{G})}_{l.l.}=\frac{|{\gamma }_{s}|P}{Q}$. Plugging the previously estimated values of *γ*_*s*_, *P* and *Q* into this lower limit, we obtain ${({\kappa }_{G})}_{l.l.}=6.16\times {10}^{+33}({T}^{2}/J\cdot m)$. Thus, we have estimated all four coupling constants needed for the Landau-Lifshitz-Ginzburg treatment of the *R3c* BFO.

The saturation magnetization (*M*_*s*_) or magnetic susceptibility (*χ*_*M*_), in general, can be readily estimated from the *M*-*H* hysteresis curve. On the contrary, Δ*M*_*eq*_ is too small^30,38^ to quantitatively discuss this effect in terms of *γ*_*s*_ and *κ*_*G*_. Thus, we have examined a variation in *χ*_*M*_. In doing this, we first consider the thermodynamic potential of the AFM subsystem. One can write the following relation by exploiting Eq. (17) for Δ*f*(*L*), Eq. (13) for the Lifshitz invariant, and Eq. (35) for the Ginzburg gradient term:

41 $${\rm{\Delta }}f(L)=\,+\frac{1}{2}{\chi }_{l}{L}^{2}+\frac{1}{4}{\xi }_{l}{L}^{4}-\frac{1}{4}{\gamma }_{q}{P}^{2}({M}^{2}-{L}^{2})+\frac{1}{2}{\kappa }_{G}{L}^{2}{Q}^{2}+{\gamma }_{s}P{L}^{2}Q\,\,$$

where ${L}^{2}={M}^{2}-4({{\boldsymbol{m}}}_{{\bf{1}}}\cdot {{\boldsymbol{m}}}_{{\bf{2}}})={M}^{2}-4{m}_{o}^{2}\,\cos \,\theta  > {M}^{2}.$ Plugging this result into Eq. (41) and adding the term, **−** ***H*** · ***M***, to Eq. (41), one can obtain the free-energy functional [Δ*f*_*H*_(*M*, *θ*)] under an external magnetic field, ***H***. Taking the dynamic equilibrium condition, i.e., ${(\partial {\rm{\Delta }}{f}_{H}(M,\theta )/\partial M)}_{P,\theta }=0,$ one can eventually derive the following relation for the inverse magnetic susceptibility:

42 $$\frac{1}{{\chi }_{M}}\equiv {(\frac{{\rm{\partial }}H}{{\rm{\partial }}M})}_{P,\theta }=\{{\chi }_{l}+3{\xi }_{l}{M}^{2}+4{\xi }_{l}{m}_{o}^{2}|\cos \,\theta |+(2{\gamma }_{s}PQ+{\kappa }_{G}{Q}^{2})\}$$

The term inside a small bracket, 2*γ*_*s*_ *PQ* + *κ*_*G*_ *Q* ^2^, is non-zero for a bulk crystal but is zero for a constrained epitaxial film. If this term is positive, the inverse susceptibility decreases or equivalently, *M*_*s*_ increases upon the transition to the homogeneous spin state in a constrained epitaxial film. On the contrary, the reverse is true if this term is negative. According to the experimental observation of *χ*_*m*_^38^, the former is true. In other words, 2*γ*_*s*_ *PQ* + *κ*_*G*_ *Q* ^2^ > 0, or equivalently, $|{\gamma }_{s}| < \frac{{k}_{G}Q}{2P}$.

According to Eq. (40), the enhanced magnetic remanence associated with the formation of epitaxially constrained film (Δ*M*_*eq*_) is determined by two material parameters, *γ*_*s*_ and *κ*_*G*_. We thus have examined the effects of these two parameters on Δ*M*_*eq*_ as a function of the wavelength (*λ*) of cycloidal SDW. In doing this, we have multiplied Δ*M*_*eq*_ by *ξ*_*l*_ *M*_*eq*(*b*)_ since Δ*M*_*eq*_ itself is too small to be experimentally evaluated. Thus, Eq. (40) is rearranged as

43 $$y\equiv \{{\xi }_{l}{M}_{eq(b)}\}\cdot {\rm{\Delta }}{M}_{eq}=+{\kappa }_{G}{Q}^{2}-|{\gamma }_{s}|PQ=\frac{4{\pi }^{2}{\kappa }_{G}}{{\lambda }^{2}}-\frac{2\pi |{\gamma }_{s}|P}{\lambda }$$

**Figure 7:**
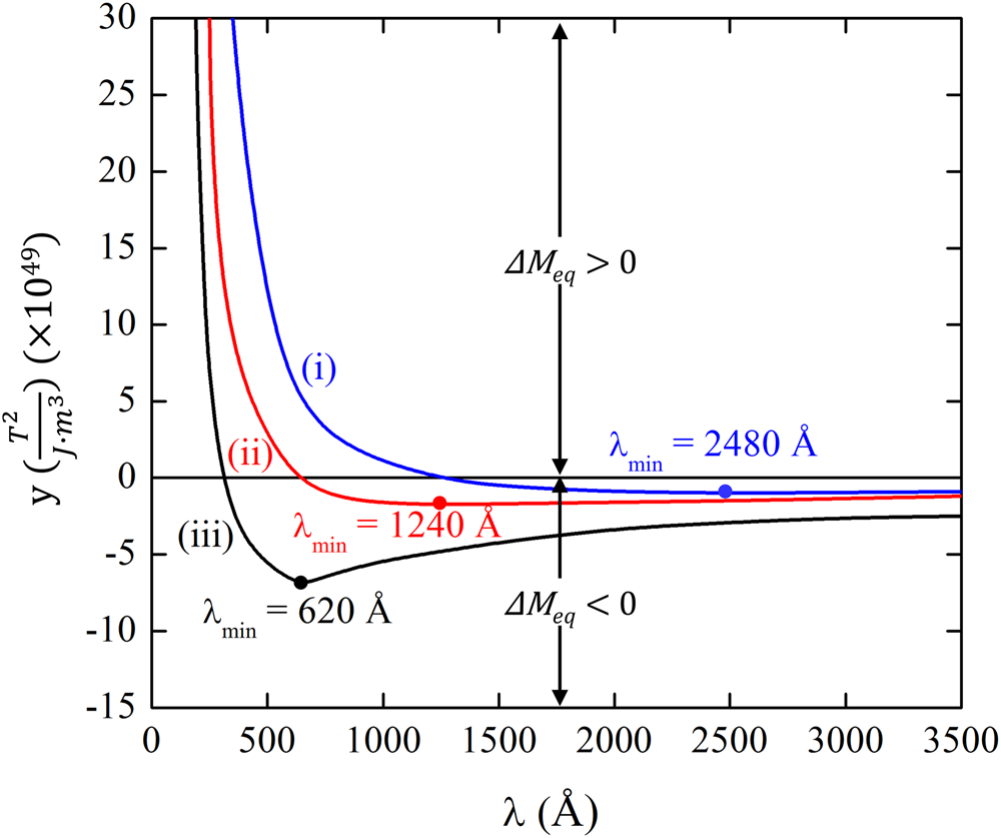
Plotting the computed *y* value, which is a measure of Δ***M***_*eq*_ as a function of the wavelength (*λ*) of SDW. The *κ*_*G*_ and |*γ*_*s*_| values corresponding to the three computed curves are: (**i**) $|{\gamma }_{s}|=7.23\times {10}^{+41}({T}^{2}/J\cdot C)$ and ${\kappa }_{G}=2{({\kappa }_{G})}_{l.l.}=12.32\times {10}^{+33}({T}^{2}/J\cdot m),$ (**ii**) $|{\gamma }_{s}|=7.23\times \,{10}^{+41}({T}^{2}/J\cdot C)$ and ${\kappa }_{G}={({\kappa }_{G})}_{l.l.}=$ $6.16\times {10}^{+33}({T}^{2}/J\cdot m),$ (**iii**) $|{\gamma ^{\prime} }_{s}|=2|{\gamma }_{s}|=14.46\times {10}^{+41}({T}^{2}/J\cdot C)$ and ${\kappa }_{G}={({\kappa }_{G})}_{l.l.}=$ $6.16\times {10}^{+33}({T}^{2}/J\cdot m)$. The same polarization (*P*) value of 0.863 (*C*/*m* ^2^) was used for all three curves. For given *κ*_*G*_ and |*γ*_*s*_| values, there exists a certain critical value of *λ* below which ${\rm{\Delta }}{M}_{eq} > 0,$ or equivalently, the latent magnetization is released upon the transition to the homogeneously canted spin state in an epitaxially constrained film. The three critical *λ*_*c*_ values are 1240 Å, 620 Å, and 310 Å for the curve (**i**), (**ii**), and (**iii**), respectively.

Thus, *y* is a measure of the enhanced magnetic remanence upon the transition to the homogeneous canted spin state in a constrained thin film. As shown in Fig. 7, *y* decreases rapidly with increasing *λ* value and reaches its characteristic minimum value, which is regardless of |*γ*_*s*_| or *κ*_*G*_ value. According to Eq. (43), *y* reaches its minimum at ${\lambda }_{min}=\frac{4\pi {k}_{G}}{|{\gamma }_{s}|P}$, explaining the computed result shown in Fig. 7. The characteristic minimum *y* value is ${y}_{min}=-\frac{|{\gamma }_{s}{|}^{2}{P}^{2}}{4{k}_{G}} < 0$. Thus, the magnitude of *y*_*min*_ is proportional to the square of |*γ*_*s*_| but is inversely proportional to *κ*_*G*_. This prediction is graphically illustrated in Fig. 7. Equation (43) further predicts that *y* becomes *0* at ${\lambda }_{c}=\frac{2\pi {k}_{G}}{|{\gamma }_{s}|P}$, which is regardless of |*γ*_*s*_|. Thus, the critical value *λ*_*c*_ corresponding to (*κ*_*G*_)_*l*.*l*._ (i.e., lower limit of *κ*_*G*_) can be deduced by plugging ${\kappa }_{G}=\frac{\,|{\gamma }_{s}|P}{Q}=6.16\times {10}^{+33}({T}^{2}/J\cdot m)$ into ${\lambda }_{c}=\frac{2\pi {k}_{G}}{|{\gamma }_{s}|P}$, yielding *λ*_*c*_ =  620 Å. This *λ*_*c*_ value corresponds to the curve (ii) in Fig. 7. In case of the *R3c* BFO, only the region with *y* ≥ 0 is experimentally meaningful since the release of the latent magnetization is observed upon the transition to the homogeneously canted spin state in epitaxially constrained BFO thin films. As shown in Fig. 7, *λ* should be smaller than a certain critical value for Δ*M*_*eq*_ > 0 (i.e., for *y* ≥ 0). Since ${\lambda }_{c}=\frac{2\pi {k}_{G}}{|{\gamma }_{s}|P}$, this critical *λ*_*c*_ value increases with *κ*_*G*_ but decreases with increasing value of |*γ*_*s*_|.

## Conclusion

We have theoretically identified the three free-energy invariants (${\rm{\Delta }}{f}_{LF},\,{\rm{\Delta }}{f}_{ms},\,{\rm{\Delta }}{f}_{G}$) that are closely related to the manifestation of the two distinct spin-coupling-induced improper polarizations by suitably exploiting the Landau-Lifshitz-Ginzburg thermodynamic potential for the *R3c* BFO. The two relevant parasitic improper polarizations are: (i) ${\rm{\Delta }}{{\boldsymbol{P}}}_{LF}$ arising from the Lifshitz gradient coupling, which can be equated with the improper polarization caused by the reverse DM interaction, Δ***P***_*DM*_, and (ii) Δ***P***_*ms*_ originating from the magnetostrictive interaction. The two improper polarizations are comparable in their magnitudes (20 *vs*. 36 *nC*/*cm* ^*2*^). The direction of the off-centering proper ferroelectric polarization (***P***_***eq***(**0**)_) is parallel to Δ***P***_*LF*_ while it is antiparallel to Δ***P***_*ms*_. We have further predicted that the magnetic susceptibility (*χ*_*m*_) increases substantially upon the transition to the homogeneous spin state in a constrained epitaxial BFO film, which accounts for the experimental observation well.

## Computational Methods

To obtain material parameters needed to quantitatively estimate three distinct polarizations and coupling constants, we have performed first-principles DFT calculations on the basis of the generalized gradient approximation (GGA)^40^ and the GGA+U method^41^ implemented with the projector augmented-wave (PAW) method^42^ using the Vienna *ab initio* simulation package (VASP)^43,44^. The Hubbard *U*_*eff*_ of 4.5 eV was chosen on the basis of empirical corrections. We explicitly treated five valence electrons for Bi (6*s* ^2^6*p* ^3^), eight for Fe (3*d* ^6^4*s* ^2^), and six for oxygen (2*s* ^2^2*p* ^4^). Actual DFT calculations were performed using (i) a 4 × 4 × 3 Monkhorst-Pack ***k***-point mesh^45^ centered at Γ for the *R3c* structure, (ii) a 500 eV plane-wave cutoff, and (iii) the tetrahedron method with the Blöchl corrections for the Brillouin-zone integrations^46^. Structural optimizations were basically performed for the 30-atoms cell which corresponds to a hexagonal unit cell. In contrast, we adopted a 2 × 2 × 1 hexagonal supercell (containing 4 unit cells) to evaluate Δ*P*_*LF*_.  The ions were relaxed until the Hellmann-Feynman forces on them were less than 0.01 eV/Å.
